# The Meeting of the A. D. A.

**Published:** 1887-09

**Authors:** 


THE MEETING OF THE A. D. A.

A large gathering was not anticipated at Niagara this year, but the number present was considerably in excess of sanguine expectations. The registry showed that about two hundred members and visitors attended. But if the meeting was not equal in point of numbers to that of last year, it excelled it in many particulars. In the first place, there was perfect harmony. The political waves were stilled, and the members present devoted themselves, in the main, to legitimate business. There was none of the wire-pulling which in 1886 converted the Association into a bear pit. The ambition of members really seemed directed toward scientific affairs, and as a consequence there was no lack of good papers, and the discussions at times were quite animated. There is no disputing the fact that in its general tone the meeting was a decided advance upon the most of its predecessors.

Dr. Allport presided with great dignity, and business matters were disposed of with precision and despatch. There was practically no opposition to the officers who were elected, and Dr. Abbott received all the votes for President, except those which might be classed as scattering. Of course Dr. Cushing was re-elected Secretary, and Dr. Keely, Treasurer.

Comparatively few dental exhibits were made. The dealers generally had anticipated a small meeting, and thought there might perhaps be opportunity to sell but few goods. The S. S. White Dental Mfg. Co., the American Dental Mfg. Co., R. S. Williams, C. Ash & Sons, of London, represented by their Mr. Sykes, of New York, were all the dental depots represented. Seabury & Johnson made a magnificent exhibition of their absorbent cotton, Hydronaphthol and other specialties, and there were exhibits of different appliances and methods, perhaps the most interesting being that of Dr. C. C. Carroll, of Meadville, Penn., who successfully demonstrated the casting of aluminum plates. Altogether, the meeting was one of interest and profit.

